# LOXL4 knockdown enhances tumor growth and lung metastasis through collagen-dependent extracellular matrix changes in triple-negative breast cancer

**DOI:** 10.18632/oncotarget.14450

**Published:** 2017-01-02

**Authors:** Sul Ki Choi, Hoe Suk Kim, Tiefeng Jin, Woo Kyung Moon

**Affiliations:** ^1^ Department of Radiology, Seoul National University Hospital, Jongno-gu, Seoul 03080, Korea; ^2^ Department of Biomedical Sciences, Seoul National University College of Medicine, Jongno-gu, Seoul 03080, Korea; ^3^ Department of Pathology and Cancer Research Center, Yanbian University Medical College, Yanji 133002, China

**Keywords:** triple-negative breast cancer (TNBC), lysyl oxidase-like 4 (LOXL4), collagen, tumor progression, overall survival

## Abstract

Lysyl oxidase (LOX) family genes catalyze collagen cross-link formation. To determine the effects of lysyl oxidase-like 4 (LOXL4) expression on breast tumor formation and metastasis, we evaluated primary tumor growth and lung metastasis in mice injected with LOXL4-knockdown MDA-MB-231 triple-negative human breast cancer cells. In addition, we analyzed overall survival in breast cancer patients based on LOXL4 expression using a public online database. In the mouse xenograft model, LOXL4 knockdown increased primary tumor growth and lung colonization as well as collagen I and IV, lysine hydroxylase 1 and 2, and prolyl 4-hydroxylase subunit alpha 1 and 2 levels. Second harmonic generation imaging revealed that LOXL4 knockdown resulted in the thickening of collagen bundles within tumors. In addition, weak LOXL4 expression was associated with poor overall survival in breast cancer patients from the BreastMark dataset, and this association was strongest in triple-negative breast cancer patients. These results demonstrate that weak LOXL4 expression leads to remodeling of the extracellular matrix through induction of collagen synthesis, deposition, and structural changes. These alterations in turn promote tumor growth and metastasis and are associated with poor clinical outcomes in triple-negative breast cancer.

## INTRODUCTION

Elevated collagen deposition and alterations in the structure of the extracellular matrix (ECM) are common in various forms of cancer [1, 2]. In particular, elevated expression and deposition of collagen I and IV as well as laminin levels has been implicated in abnormal stiffness of the ECM [3]. Aberrant expressions of lysyl oxidase (LOX) family genes, which catalyze collagen cross-link formation, and the procollagen-lysine 2-oxyglutarate 5-dioxygenase (PLOD) and prolyl 4-hydroxylase α subunit (P4HA) genes, which mediate collagen lysine hydroxylation, also alter the structure of the ECM [4–10].

Levels of lysyl oxidase-like 4 (LOXL4) are much lower than levels of other LOX family members in various normal tissues [11]. In addition, the associations between aberrant LOXL4 expression and its pathophysiological effects in cancer are similar to those observed for other LOX family members [12–17]. However, previous reports have obtained conflicting results regarding the effects of LOXL4 in cancer. In bladder cancer, LOXL4 suppresses tumors by inhibiting the oncogenic signaling pathway [16]. In contrast, LOXL4 promotes aggressive tumor progression and metastasis in colorectal and oral cancers [17, 18]. In breast cancer, LOXL4, LOX, and LOXL2, which are expressed in a hypoxia-inducible factor 1-dependent manner, recruit bone marrow-derived cells and facilitate colonization of the lungs [19]. These conflicting results might be explained by differences in the cellular context among cancers that might influence whether LOXL4 acts as a tumor suppressor or a metastasis promoter. Although many studies have been conducted to elucidate the role of LOXL4 in cancer, the precise mechanisms by which LOXL4 suppresses tumors or promotes metastasis in breast cancer remain largely unknown. Clinical studies have shown that high LOX and LOXL2 expression are correlated with increased metastasis and poor survival in triple negative breast cancer (TNBC) patients [20, 21]. Interestingly, a recent report revealed that, among the LOX family members, LOXL4 mRNA levels alone were higher in cancer tissues from TNBC patients than in those from estrogen receptor-positive breast cancer patients [22]. Although these reports suggest that LOXL4 expression might affect clinical outcomes in TNBC patients, to the best of our knowledge, no studies have directly examined this relationship. Here, we injected mice with LOXL4-knockdown MDA-MB-231 cells, which are aggressive TNBC cells, to investigate the roles of LOXL4 in primary tumor growth and metastasis in a xenograft model. In addition, we evaluated the clinical significance of LOXL4 in human breast cancer patients using a public database with overall survival (OS) information.

## RESULTS

### LOXL2, LOXL3, and LOXL4 expression are higher in MDA-MB-231 cells than in other human breast cancer cells

**Figure 1:**
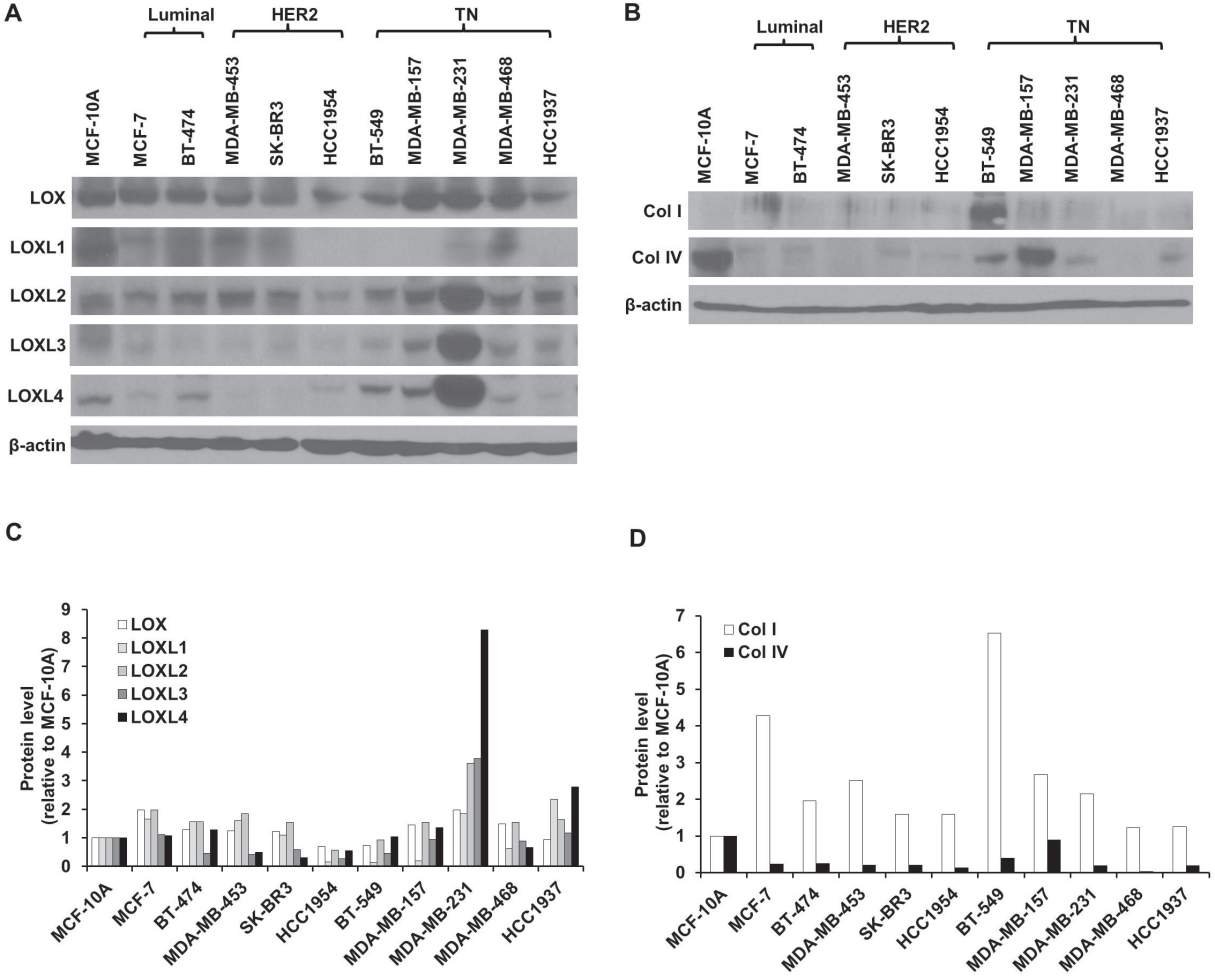
LOX family member, collagen I, and collagen IV expression in various breast cancer cells **A** and **B**. Western blotting analysis of LOX family (LOX and LOXL1-4), collagen I, and collagen IV protein expression in normal breast epithelial cells (MCF-10A) and luminal (MCF-7, BT-474), HER2 (MDA-MB-453, SK-BR3, and HCC1954), and TN (BT-549, MDA-MB-157, MDA-MB-231, MDA-MB-468, and HCC1937) breast cancer cell lines. **C** and **D**. LOX family, type I procollagen (collagen I), and collagen IV protein expression in breast cancer cells relative to MCF-10A cells.

We measured the expression of LOX and LOXL1-4 in a total of 10 human breast cancer cell (hBCC) lines that are classified as either luminal (MCF-7, BT-474), HER2 (MDA-MB-453, SK-BR3, HCC1954), or TN (BT-549, MDA-MB-157, MDA-MB-231, MDA-MB-468, HCC1937) subtype, as well as in normal breast epithelial cells (MCF-10A). As shown in Figure 1A and 1C, LOX and LOXL2 expression were abundant in all of the hBCCs and in the normal breast epithelial cells. LOXL1 was highly expressed in both the luminal subtype cells (MCF-7, BT-474) and in two of the three HER2 subtype cells (MDA-MB-453, SK-BR3), but was expressed at very low levels in HER2 subtype HCC1954 cells and in the TN subtype cells (BT-549, MDA-MB-157, MDA-MB-231, MDA-MB-468, HCC1937). In contrast, LOXL3 and LOXL4 expression was higher in the TN subtype cells (BT-549, MDA-MB-157, MDA-MB-231, HCC1937) than in the other subtypes. Furthermore, LOXL2, LOXL3, and LOXL4 expression were higher in MDA-MB-231 cells than in the other TN cells (BT-549, MDA-MB-157, MDA-MB-468, HCC1937). In addition, LOXL4 expression was higher in MDA-MB-231 cells than the expression of the other LOX family members that were elevated in TN cells. We also examined type I procollagen (collagen I) and collagen IV expression in the various hBCC types. Collagen I expression was higher in BT-549 cells, and collagen IV expression was higher in MCF-10A and MDA-MB-157 cells, than in the other hBCCs (Figure 1B and 1D).

### LOXL4 knockdown promotes migration

**Figure 2:**
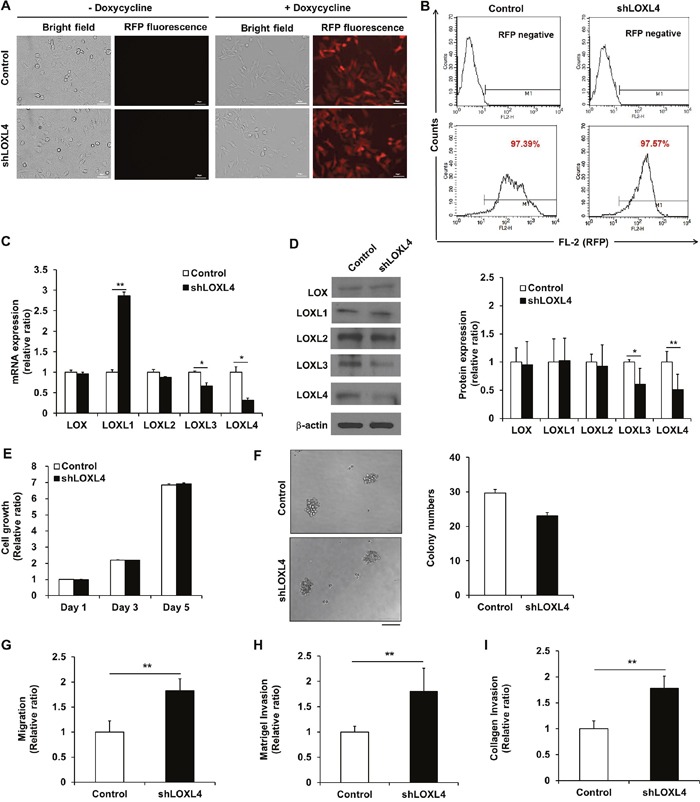
Establishment and characterization of LOXL4 knockdown MDA-MB-231 cells **A**. Fluorescence images of doxycycline-inducible red fluorescent protein expression in MDA-MB-231 cells transduced with lentivirus encoding both RFP and either the non-silencing control vector or shLOXL4. Scale bar: 50 μm. **B**. Flow cytometric analysis of the percentage of RFP-positive control or LOXL4-knockdown MDA-MB-231 cells. **C**. Quantitative real-time RT-PCR of LOX and LOXL1-4. **D**. Western blotting analysis of LOX family member protein expression. **E**. MTT assay for analysis of cell proliferation. **F**. Single cell colony formation assay. Scale bar: 100 μm. **G**. Trans-well migration assay for the analysis of cell migratory capacity. **H** and **I**. Trans-well invasion assays for the analysis of cell invasion capacity. All experiments were performed at least in triplicate; means ± standard deviation of all experiments are shown. *P < 0.05. **P < 0.001. Scale bar: 50 μm.

Fluorescence microscopy confirmed that the lentiviral vector facilitated doxycycline-dependent inducible RFP expression (Figure 2A), and flow cytometry revealed that the efficiency of lentivirus-mediated RFP gene transfer was over 97% (Figure 2B). Real-time RT-PCR revealed that lentiviral transduction of LOXL4 shRNA decreased LOXL4 mRNA levels (0.32 ± 0.05-fold) compared to the control (Figure 2C, *P* = 0.002). LOXL3 and LOXL4 are identical except for three amino acids; due to this similarity, LOXL3 mRNA levels also decreased 0.67 ± 0.08-fold in LOXL4-knockdown cells compared to the control (Figure 2C, *P* = 0.038). Interestingly, LOXL1 mRNA levels increased 2.87 ± 0.09-fold in LOXL4-knockdown cells compared to the control (Figure 2C, *P* = 0.0002). Consistent with the real-time RT-PCR results, Western blot analysis revealed that LOXL3 (0.61 ± 0.28-fold, *P* = 0.015) and LOXL4 (0.51 ± 0.27-fold, *P* < 0.001) protein levels were significantly decreased in LOXL4-knockdown cells compared to the control cells (Figure 2D). However, despite the increase observed in LOXL1 mRNA levels, LOXL4 knockdown did not change LOXL1 protein levels (Figure 2D). After the LOXL1 precursor protein is synthesized, it undergoes post-translational processing by bone morphogenetic protein 1 [23, 24]; this may explain the inconsistency observed in LOXL4-knockdown-induced changes in LOXL1 mRNA and protein levels.

The growth rates and colony forming capacity observed in LOXL4-knockdown MDA-MB-231 cells were similar to those of control MDA-MB-231 cells (Figure 2E and 2F). The migratory ability of LOXL4-knockdown cells increased 1.82 ± 0.24-fold in the trans-well migration assay (Figure 2G, *P* < 0.001). The invasive ability of LOXL4-knockdown cells also increased by 1.80 ± 0.46-fold in the Matrigel™ matrix-coated trans-well assay (Figure 2H, *P* = 0.0001) and 1.78 ± 0.23-fold in the type I rat tail collagen-coated trans-well assay (Figure 2I, *P* < 0.001) compared to control cells. Cell characterization assays after LOXL4 knockdown were also conducted using MCF-7 (luminal subtype) and BT-549 (TN subtype) breast cancer cells. LOXL4-knockdown and control MCF-7 cells had similar growth rates (Supplementary Figure 1C), but growth rates decreased in LOXL4-knockdown BT-549 on days 3 and 5 after cell seeding compared to control BT-549 cells (*P* < 0.001, Supplementary Figure 2C). The colony forming capacities of LOXL4-knockdown MCF-7 and BT-549 cells were similar to those of the respective control cells (Supplementary Figures 1D and 2D). Migratory capacity did not differ between LOXL4-knockdown and control MCF-7 cells (Supplementary Figure 1E-G), but migratory and invasive abilities increased in LOXL4-knockdown BT-549 in the trans-well migration (1.31 ± 0.07-fold, *P =* 0.002) and the Matrigel™ matrix (1.87 ± 0.34-fold, *P* = 0.001), and collagen (1.85 ± 0.20-fold, *P* < 0.001) trans-well invasion assays compared to control BT-549 cells (Supplementary Figure 2E-G).

### LOXL4 knockdown promotes primary tumor growth and lung metastatic tumor formation

**Figure 3:**
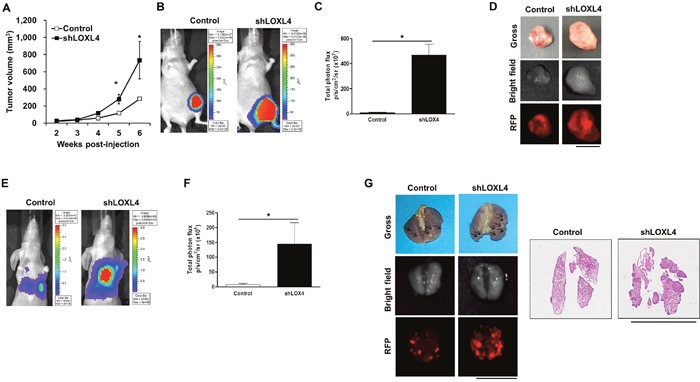
LOXL4 knockdown increased primary tumor growth and lung metastasis of MDA-MB-231 breast cancer cells **A**. Changes in tumor volume after injection of MDA-MB-231 cells transduced with control or shLOXL4. **B**. Representative bioluminescence images of the tumors obtained with the IVIS system on day 41 after cancer cell injection. **C**. Total flux values (p/s/cm^2^/sr) measured in bioluminescence images (day 41) of the primary tumors. Control and shLOXL4, n = 4 and 5, respectively. **D**. Images of the final lung gross appearance and doxycycline-induced RFP in primary tumors. **E**. Representative bioluminescence images of tumor burdens in lungs obtained on day 56 after tail vein injections of cancer cells. n = 5. **F**. Total flux (p/s/cm^2^/sr) measured in bioluminescence images of the metastatic lung burden (day 56). **G**. Images of the final lung gross appearance, doxycycline-induced RFP in lung metastatic nodules, and H&E staining. Scale bar: 1 cm.

We next investigated whether LOXL4 knockdown increased primary tumor growth and metastatic tumor formation *in vivo*. In an orthotopic xenograft model (n = 4 and 5 for the control and LOXL4-knockdown groups, respectively), LOXL4 knockdown increased tumor volumes in the 5^th^ and 6^th^ weeks after cancer cell injection compared to the control (Figure 3A, *P* = 0.008 and *P* = 0.040, 5^th^ and 6^th^ weeks, respectively). Bioluminescence images (BLI) and associated total flux values (p/s/cm^2^/sr) were obtained using the IVIS system just before mice were sacrificed. As expected, *in vivo* BLI signals were stronger in LOXL4-knockdown tumor sites (6.13×10^7^ p/s/cm^2^/sr) than in control tumor sites (468.54×10^7^ p/s/cm^2^/sr) (Figure 3B–3C, *P* = 0.035). The *g*ross appearance and *ex vivo* RFP fluorescence images showed bigger primary tumors from in LOXL4-knockdown mice than those from the control mice (Figure 3D). In the lung metastasis model, BLI signals indicated that the lung nodule formation ability of LOXL4-knockdown cells (15.05×10^5^ p/s/cm^2^/sr) was increased compared with that of control cells (108.77×10^5^ p/s/cm^2^/sr) (*P* = 0.014, Figure 3E-3F). Images of the gross appearance of the lungs and of RFP fluorescence detection also clearly confirmed that LOXL4-knockdown increased numbers of lung tumor nodules compared to the control (Figure 3G).

### LOXL4 knockdown increases collagen I and IV, PLOD1, PLOD2, P4HA1, and P4HA2 expression in xenograft tumor tissues

**Figure 4:**
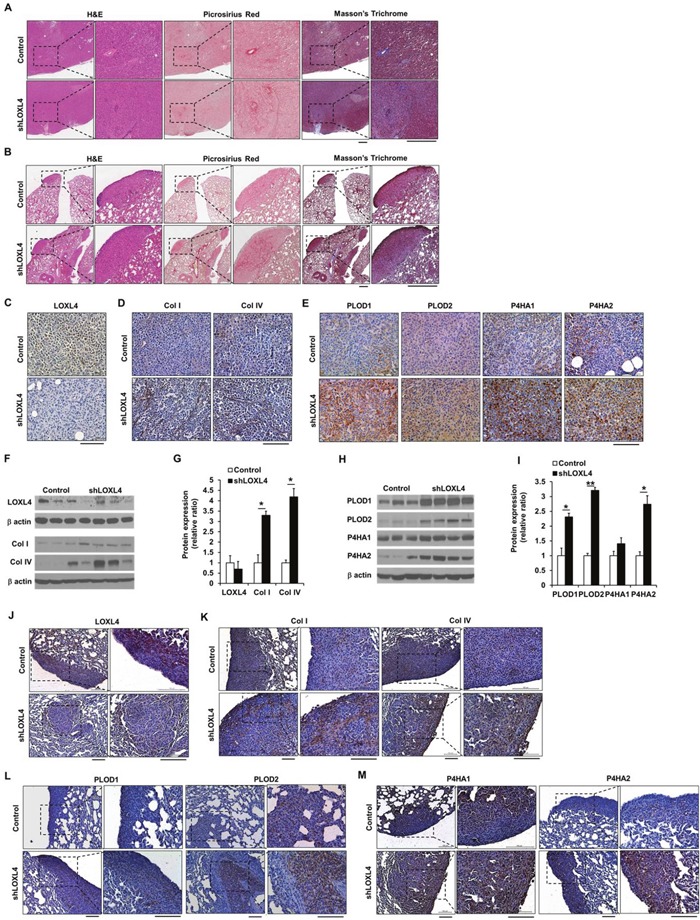
LOXL4 knockdown increases collagen synthesis and deposition **A**. Images of H&E-, Picrosirius red-, and Masson's trichrome-stained sections of primary tumors resulting from injection of control and LOXL4-knockdown cells. Scale bar: 400 μm. **B**. Images of H&E-, Picrosirius red-, and Masson's trichrome-stained lung sections after injection of control and LOXL4-knockdown cells. Scale bar: 400 μm. **C** and **D**. Representative immunohistological (IHC) images of LOXL4, collagen I, and collagen IV staining in primary tumors. Scale bar: 100 μm. **E**. Representative IHC images of PLOD1-2 and P4HA1-2 staining in primary tumors. Scale bar: 100 μm. **F**. Representative images of Western blots for LOXL4, type I procollagen (collagen I), and collagen IV. **G**. Densitometric quantification of LOXL4, type I procollagen (collagen I), and collagen IV expression in tumors. **H**. Western blotting analysis of PLOD1-2 and P4HA1-2 expression in primary tumors. **I**. Densitometric quantification of PLOD1-2 and P4HA1-2 expression in tumors. **J** and **K**. Representative IHC images of LOXL4, collagen I, and collagen IV staining in the lungs. Scale bar: 100 μm. **L** and **M**. Representative IHC images of PLOD1-2 and P4HA1-2 staining in the lungs. Scale bar: 200 μm. *P < 0.05. **P < 0.001.

To investigate whether LOXL4 knockdown altered the structure of the ECM in tumor tissues, Picrosirius red and Masson's trichrome staining were conducted. Strongly stained regions were observed within both primary tumors and lung nodules (Figure 4A–4B). Histological analysis of primary tumors confirmed that LOXL4 expression was downregulated in LOXL4-knockdown tumors (Figure 4C). Type I procollagen (Collagen I) and IV immunohistochemical (IHC) staining intensities were stronger in LOXL4 knockdown tumors than in control tumors (Figure 4D). LOXL4 knockdown also increased PLOD1-2 and P4HA1-2 levels compared to controls (Figure 4E). Consistent with the IHC results, western blots revealed a trend towards decreased LOXL4 protein expression in LOXL4-knockdown tumors, although this difference was not statistically significant (Figure 4F–4G). Type I procollagen (collagen I) and IV expression levels increased 3.30 ± 1.40-fold (*P* = 0.01) and 4.19 ± 0.46-fold (*P* = 0.047), respectively, in LOXL4-knockdown tumors (Figure 4F–4G). PLOD1 (2.31 ± 1.13-fold, *P* = 0.002), PLOD2 (3.21 ± 0.10-fold, *P* < 0.001), and P4HA2 (2.74 ± 0.29-fold, *P* = 0.048) expression also increased in LOXL4-knockdown tumors compared to control tumors (Figure 4H–4I). In addition, PLOD1 (1.29 ± 0.06-fold, *P* = 0.047) and PLOD2 (1.49 ± 0.18-fold, *P* = 0.013) expression also increased in LOXL4-knockdown MDA-MB-231 cells relative to control cells (Supplementary Figure 4). IHC staining of collagen I and IV, PLOD1-2, and P4HA1-2 in lung metastatic tissues revealed that LOXL4 expression was suppressed in LOXL4-knockdown lung tumors (Figure 4J). Collagen I and IV staining was stronger in LOXL4-knockdown lung tumors than in control tumors (Figure 4K); PLOD1 and P4HA1-2 staining were also strong in LOXL4-knockdown lung tumors (Figure 4L–4M).

### LOXL4 knockdown promotes thickening of collagen fibers

**Figure 5:**
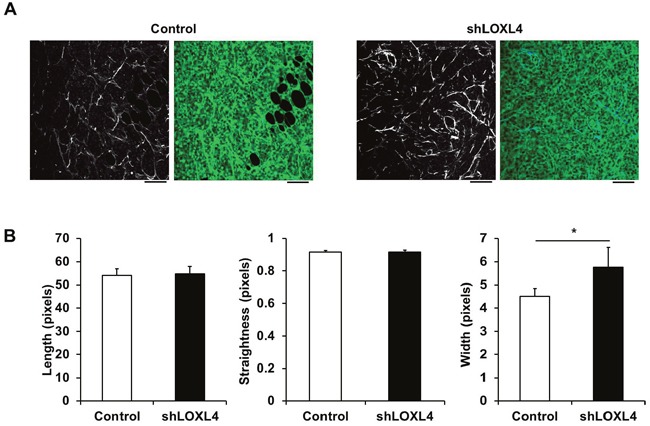
Second harmonic generation (SHG) imaging of control and LOXL4-knockdown primary tumor tissues **A**. Representative SHG images alone (left) and overlaid with the fluorescence images of control and LOXL4-knockdown primary tumor tissues (right). **B**. Quantification of collagen fiber lengths, straightness, and widths in control and LOXL4-knockdown primary tumor tissues. n = 5. *P < 0.05. Scale bar: 100 μm.

We then used SHG imaging to examine whether LOXL4 knockdown altered collagen fiber structure and organization. Collagen fiber quantification was conducted using CT-FIRE, an open-source software package that was developed to automatically quantify individual collagen fibers in SHG images (http://loci.wisc.edu/software/ctfire) [25]. Fiber lengths and widths were calculated as pixel values. Straightness was calculated by dividing the distance between each fiber's end points by the fiber path; the scale for this metric was 0-1, with 1 indicating a straight line. Although collagen fiber length and straightness did not differ between control and LOXL4-knockdown tissues, collagen fiber width was elevated in LOXL4-knockdown tumors compared to control tumors (Figure 5A–5B, *P* = 0.041).

### Low LOXL4 and high collagen expression are associated with poor overall survival

**Figure 6:**
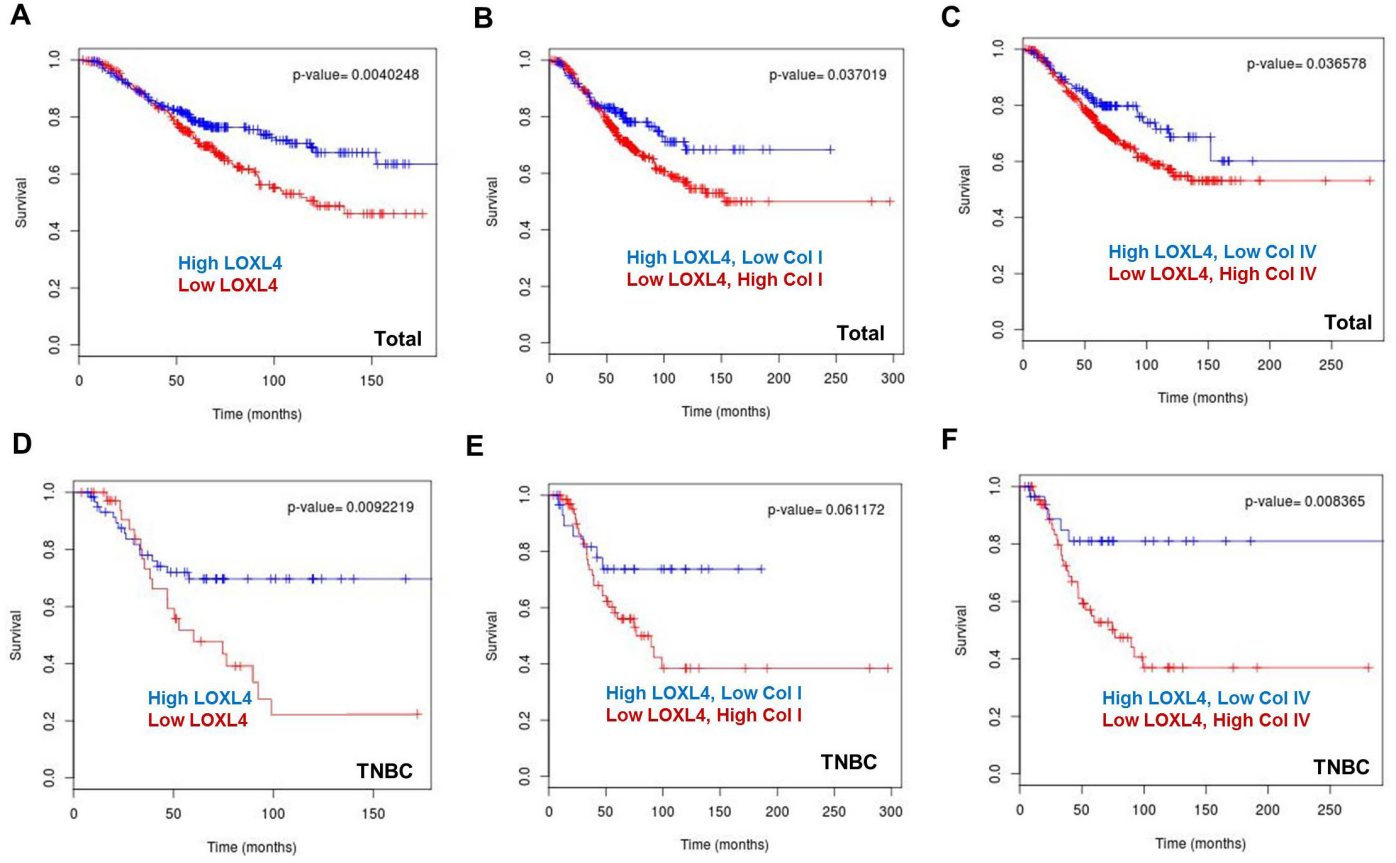
Kaplan-Meier plots of breast cancer patient survival based on LOXL4, collagen I, and collagen IV expression in the BreastMark dataset **A**. Low LOXL4 expression was associated with poor overall survival (OS) in breast cancer. n = 584, number of events = 169, Hazard ratio (HR) = 0.6395 (0.4704-0.8694), score (log rank) test = 8.28, *P* = 0.004. **B**. The combination of low LOXL4 and high collagen I expression was associated with poor OS in breast cancer. n = 584, number of events = 169, HR = 0.6718 (0.4612-0.9787), score (log rank) test = 0.08, *P* = 0.037. **C**. The combination of low LOXL4 and high collagen IV expression was associated with poor OS in breast cancer. n = 584, number of events = 169, HR = 0.6619 (0.4483-0.9774), score (log rank) test = 15.36, *P* = 0.036. **D**. Low expression of LOXL4 was associated with a poor OS in triple negative breast cancer (TNBC). n = 101, number of events = 36, HR = 0.427 (0.2207-0.8259), score (log rank) test = 6.78, *P* = 0.009. **E**. The combination of low LOXL4 and high collagen I expression showed a trend towards association with poor OS in TNBC. n = 101, number of events = 36, HR=0.4621 (0.2018-1.058), score (log rank) test = 3.5, *P* = 0.061. **F**. The combination of low LOXL4 and high collagen IV expression was associated with a poor OS in TNBC. n = 101, number of events = 36, HR = 0.3006 (0.1166-0.7749), score (log rank) test = 6.96, *P* = 0.008.

The BreastMark website was used to explore the association between LOXL4 and collagen I and IV expression and OS in breast cancer patients. A total of 584 samples from 11 datasets were analyzed, including 169 events from BreastMark, and the samples were separated into high and low LOXL4 expression groups. Kaplan-Meier plots revealed that OS was significantly shorter in the low LOXL4 level group (Figure 6A, *P* = 0.004, HR = 0.6395). Interestingly, OS was poorest in breast cancer patients with both low LOXL4 levels and high collagen I or IV expression (Figure 6B–6C, *P* = 0.037, HR = 0.6718 and *P* = 0.037, HR = 0.6619, respectively). We also analyzed OS in a set of 101 patients that included 36 who were diagnosed with TNBC using the PAM50 assay. Low LOXL4 expression was also associated with poor OS in these patients (Figure 6D, *P* = 0.009, HR = 0.427). While the trend towards an association between the combination of low LOXL4 and high collagen I expression and poorer OS did not reach significance (Figure 6E, *P* = 0.061, HR=0.4621), the low LOXL4 and high collagen IV expression combination was significantly associated with poorer OS in TNBC patients (Figure 6F, *P* = 0.008, HR = 0.3006).

## DISCUSSION

In the present study, we demonstrated that knockdown of LOXL4 expression promoted primary tumor growth and lung metastasis in MDA-MB-231 cell xenograft models of breast cancer. These increases in tumor growth and metastasis were accompanied by alterations in the synthesis, deposition, structure of collagen, and specifically by an increase in bundle thickness. Furthermore, we found that low LOXL4 expression was associated with poorer overall survival in breast cancer patients. To the best of our knowledge, this is the first report to demonstrate a link between low LOXL4 expression and increased progression in TNBC.

First, we evaluated the expression of the LOX family members, as well as collagen I and IV, in a diverse set of breast cancer cell lines. LOXL4 expression was higher in MDA-MB-231 cells, which are aggressive TNBC cells, than in cells belonging to the luminal and HER2 subtypes. We then examined the effects of shRNA-mediated LOXL4 knockdown in MDA-MB-231 cells; knockdown of LOXL4 increased the migratory and invasive abilities of these cells. While Kirschmann *et al*. reported that LOX and LOXL2 expression were most strongly associated with invasive potential in both highly invasive and metastatic breast cancer cell lines [26], our results indicate that LOXL4 also suppresses invasion and migration in MDA-MB-231 cells. These findings are inconsistent with a previous report that high LOXL4 mRNA levels may promote the transition from the solid to the effusion state in breast carcinomas [27]. We also found that LOXL4 expression differed among the hBCCs based on the breast cancer subtype to which they belonged. It is therefore likely that the role of LOXL4 may depend on heterogenous traits that vary in different types of breast cancer. Additional studies are needed to investigate the different roles of LOXL4 in breast cancer subtypes, particularly in TNBC.

Although recent studies have examined the role of LOXL4 in breast cancer, the explanation for the seemingly contradictory results obtained in cell lines and in xenograft models remains unclear. In our orthotopic primary tumor and lung metastasis models, we showed that LOXL4 knockdown increased primary tumor growth and metastasis in MDA-MB-231 cells. The authors of the one other study using LOXL4-knockdown MDA-MB-231 cells concluded that LOXL4 knockdown did not affect primary MDA-MB-231 cell tumor growth, but decreased spontaneous metastasis of MDA-MB-231 cells to the lungs in SCID and non-obese diabetic-SCID mice. The recruitment of CD11b-positive bone marrow-derived cells to sites of metastasis in the lungs was thought to underlie this effect [19]. These results, which contradict the findings of the present study, may be due to the use of different lung metastasis models, as well as differences in the tumor microenvironments of the host mice.

In this study, LOXL4 knockdown increased collagen I and IV accumulation in both primary and lung tumor tissues in the xenograft models. PLOD1-2 and P4HA1-2, enzymes that are critical for collagen synthesis, may be predictive biomarkers for human cancer progression and metastasis [8–10]. Intriguingly, we found that PLOD1-2 and P4HA1-2 expression were elevated in LOXL4-knockdown primary and lung tumors. The increases in collagen I and IV expression in LOXL4-knockdown tumors might be associated with this increase in PLOD1-2 and P4HA1-2 expression, which is likely regulated by LOXL4-mediated intracellular signaling. In addition, NADPH oxidase (NOX) family genes, which also have pro-fibrotic effects, regulate the ECM and collagen synthesis in response to other signaling mechanisms, such as TGF-β and reactive oxygen species [28]. Although we did not investigate the effects of interactions between LOXL4 and NOX on collagen production in this study, NOX might also contribute to the LOXL4 knockdown-induced increase in collagen synthesis in breast cancer cells.

Straightened and aligned collagen fiber bundles were correlated with poor disease-specific and disease-free survival in an evaluation of the relationship between tumor-associated collagen signature-3 (TACS-3) and long-term survival rates in breast cancer patients [29]. Here, we found that collagen bundles were thicker in LOXL4-knockdown tumors than in control tumors, but no changes in the linearization or the length of the collagen fibers were found. This indicates that increased collagen bundle thickness in LOXL4-knockdown tumors might have accelerated primary tumor growth and lung metastasis in the MDA-MB-231 xenograft model.

Although extensive preclinical research has advanced our understanding of the mechanisms by which the LOX family increases fibrosis in the ECM, few studies have linked LOXL4 expression with clinical outcomes in breast cancer patients. A recent study found that LOXL4 was downregulated in hepatocellular carcinoma, and this downregulation was closely correlated with worse clinical outcomes [30]. Similarly, based on an OS analysis using data from the public database at the BreastMark website, we found that low LOXL4 expression was also associated with poor OS in breast cancer patients, suggesting that LOXL4 expression could be a useful prognostic marker in breast cancer. Moreover, this relationship between low LOXL4 expression and poor clinical outcome was strongest in patients with TNBC. Finally, OS was worst in TNBC patients with both low LOXL4 and high collagen IV expression.

In this study, we demonstrated that low LOXL4 expression is associated with increases in the progression of aggressive TNBC. However, because LOXL4 expression is also associated with other risk factors, particularly collagen levels and structural changes in the ECM, future studies with larger patient populations are needed to determine whether low LOXL4 levels can serve as an independent prognostic factor in TNBC.

## MATERIALS AND METHODS

### Cell lines

The human breast cancer cell lines MCF-7, BT-474, MDA-MB-453, SK-BR3, HCC1954, BT-549, MDA-MB-157, MDA-MB-231, MDA-MB-468, and HCC1937, and normal breast epithelial cells MCF-10A, were obtained from ATCC (Manassas, VA, USA) or the Korean Cell Line Bank (Seoul, Korea). The breast cancer cells used in this study were authenticated and validated by DNA fingerprinting (AmpFLSTR identifier PCR Amplification kit), which was conducted by the Korean Cell Line Bank or provided by the distributors.

### Lentiviral transduction

Viral vectors containing either the TRIPZ-inducible lentiviral non-silencing shRNA control as a negative control vector or a TRIPZ-inducible LOXL4 shRNA (shLOXL4: clone ID V2THS_138014) construct and the red fluorescent protein (RFP) construct were purchased from GE Dharmacon (Pittsburgh, PA, USA). Lentiviral transduction was conducted according to the manufacturer's instructions. RFP-positive cells were selected from cultures maintained in medium containing 0.5-3 μg/mL puromycin for 2 weeks; selected cells were then sorted using a FACSCalibur flow cytometer (BD Biosciences, Franklin Lakes, NJ, USA) and cultured in medium without puromycin. Cancer cells that stably expressed RFP and either the non-silencing shRNA or the LOXL4 shRNA, denoted control cells and shLOXL4 cells, respectively, were generated for use in all subsequent studies. Viral vectors containing the luciferase and green fluorescent protein (GFP) constructs were also transduced for the animal study using the same procedure described above. GFP-positive cells were then selected and sorted using a FACSCalibur flow cytometer.

### RNA isolation and quantitative real-time PCR

Total RNA was isolated using TRIzol Reagent (Invitrogen, Carlsbad, CA, USA) and was reverse-transcribed using random hexamers and Superscript III reverse transcriptase. cDNAs were synthesized using M-MLV reverse transcriptase (New England Biolabs, Ipswich, MA, USA) and random primers. mRNA levels were measured in control and shLOXL4 cells using the quantitative real-time method and the following primer sets: LOX (174 bp) F, GTTCCAAGCTGGCTACTC, and R, GGGTTGTCGTCAGAGTAC; LOXL1 (244 bp) F, CAGACCCCAACTATGTGCAA, and R, ATGCTGTGGTAATGCTGGTG; LOXL2 (239 bp) F, GGAAAGCGTACAAGCCAGAG, and R, GCACTGG ATCTCGTTGAGGT; LOXL3 (162 bp) F, ATGGGTGCT ATCCACCTGAG, and R, GAGTCGGATCCTGGTC TCTG; LOXL4 (165 bp) F, ACCGAAGACAAAGCC ACAAC, and R, CACACGACACTGGCAGAGAT; and β-actin (335 bp) F, TTCCTGGGCATGGAGTCCTGTGG, and R, CGCCTAGAAGCATTTGCGGTGG. Relative gene expression was determined using an ABI 7500 real-time polymerase chain reaction (PCR) instrument (Applied Biosystems, South San Francisco, CA, USA) with pre-optimized conditions. PCR reactions were performed in triplicate. Expression ratios were calculated as the normalized threshold cycle (Ct) difference between the control and samples after adjustment for amplification efficiency relative to expression of the housekeeping gene β-actin.

### Cell viability and proliferation assay

*In vitro* cell viability and proliferation were assessed using the 3-(4, 5-dimethylthiazol-2-yl)-2,5-diphenyl tetrazolium bromide (MTT) assay. One, 3, and 5 days after cell seeding, MTT solution (final concentration 1 mg/mL) was added, and cells were incubated for 1 h. The medium was then carefully removed and the dimethyl sulfoxide was added to each well. The amount of formazan crystals formed by viable cells was determined by measuring absorbance at 540 nm with a spectrophotometer (GE Healthcare, Piscataway, NJ, USA).

### Single cell colony formation assay

Breast cancer cells were trypsinized to generate single cell suspensions and seeded at a low density (500 cells/well in 96-well plates) in Dulbecco's Modified Eagle's Medium-F12 supplemented with B-27 (Invitrogen), 20 ng/mL epidermal growth factor (BD Biosciences), 10 ng/mL leukemia inhibitory factor (Invitrogen), and 20 ng/mL basic fibroblast growth factor (BD Biosciences). Seven to 14 days later, average numbers of colonies per well were calculated. These experiments were performed in triplicate.

### Migration and invasion assays

To assess cell migratory ability, 5 × 10^4^ cells were suspended in 100 μL of medium containing no FBS and were deposited in the upper chambers of a trans-well plate with 8.0-μm pores (BD Biosciences) and non-coated membranes. For the invasion assays, 5 × 10^4^ cells were plated in 2% Matrigel™ (BD Biosciences) basement membrane matrix- or 5 μg/cm^2^ type I rat tail collagen-coated upper chambers of trans-well plates with 8.0-μm pores. The lower chambers were filled with 600 μL of medium supplemented with 10% FBS, and the cells were incubated for between 24-48 h at 37 °C depending on the cell line. Cells that migrated from the upper chambers to the bottom chambers were stained using a crystal violet solution (0.5% crystal violet in 20% methanol) for 5 min. Unbound crystal violet was removed by rinsing with distilled water. The cells were then air dried, after which the crystal violet was eluted from the cells using a 1% sodium dodecyl sulfate (SDS) solution. The absorbance of the crystal violet was measured at 550 nm using a spectrophotometer (GE Healthcare).

### Western blotting

Cells were lysed in RIPA buffer (Sigma, St. Louis, MO, USA), and proteins were separated using SDS-polyacrylamide gel electrophoresis and transferred to nitrocellulose membranes. The membranes were blocked using 5% skim milk in Tris-buffered saline containing 0.1% Tween-20 and incubated with primary antibodies directed against LOX, LOXL1, LOXL2, LOXL3 (Santa Cruz Biotechnology, Santa CruzCA, USA), LOXL4 (Abcam, Cambridge, MA, USA), Collagen I (Novus, Littleton, CO, USA), Collagen IV (Abcam), PLOD1, PLOD2, P4HA1, P4HA2 (Novus), or β-actin (Sigma) overnight at 4°C. The membranes were then incubated with horseradish peroxidase (HRP)-conjugated secondary antibodies (Santa Cruz Biotechnology). The blotted membranes were visualized using enhanced chemiluminescence reagents (GE Healthcare). Western blot quantification was performed using ImageJ software.

### Animals and xenograft tumor models

All animal experiments were approved by the Seoul National University Hospital Biomedical Research Institute Animal Care and Use Committee (IACUC). 5- to 6-week-old female BALB/c nude mice were used. For the orthotopic xenograft tumor model, a total of 5 × 10^6^ breast cancer cells per mouse were resuspended in Matrigel™ and injected into the 4^th^ mammary glands of mice. The mice were separated into two groups of 5 mice each; group 1 received cells expressing non-silencing shRNA (control), while group 2 received cells expressing inducible LOXL4 shRNA (shLOXL4). No tumors grew in one of the 5 control group mice; only data from the remaining 4 control group mice were used. The lung metastatic tumor models were created by administering a total of 5 × 10^5^ breast cancer cells per mouse into the tail veins of mice. The mice were separated into the same two groups described above; each group again had 5 mice. To induce LOXL4 knockdown *in vivo*, normal drinking water was replaced with 3% sucrose (added to increase palatability) with 2 mg/mL doxycycline (Sigma) and changed every 2-3 days. Primary tumor volume after implantation was measured using calipers. To determine xenograft tumor volumes, a modified ellipsoidal formula for volume (volume = 1/2[length x width^2^]) was used in which the length was the largest longitudinal diameter of the tumor and the width was the largest transverse diameter of the tumor.

### Bioluminescent and RFP fluorescence imaging

To monitor primary tumor growth and metastasis non-invasively, bioluminescent imaging (BLI) was conducted on the IVIS 100 system (Caliper Life Sciences, Hopkinton, MA, USA). Once each week, the firefly luciferase substrate D-luciferin (Promega, San Luis Obispo, CA, USA) was injected intraperitoneally at a dose of 150 mg/kg, and images of the tumor and lung areas were acquired 10 minutes later to evaluate peak intensities. The sum of all detected photon counts within oval-shaped regions of interest (ROI, tumor or lung) was quantified in units of mean photons per second per square centimeter per steradian (p/s/cm^2^/sr) using Living Image® software (Caliper Life Sciences). For *ex vivo* RFP fluorescence imaging, mice were anesthetized and primary tumors and lung tissues were excised. *Ex vivo* RFP fluorescence images were obtained using the Maestro imaging system (CRi, Woburn, MA, USA); spectral fluorescence images consisting of autofluorescence spectra and spectra from RFP were then unmixed based on their spectral patterns using Maestro software (CRi).

### Histological analysis

Histological analyses of primary tumors and cancer nodules in the lungs were performed. The primary tumors and lungs were removed 6 or 10 weeks after control or shLOXL4 cells were injected. Hematoxylin and eosin (H&E), Masson's trichrome, or Picrosirius red stainings, and immunostaining using primary LOXL1 (Santa Cruz Biotechnology), LOXL4 (Abcam), Collagen I (Novus), Collagen IV (Abcam), PLOD1, PLOD2, P4HA1, and P4HA2 (Novus) antibodies and HRP-conjugated secondary antibodies (Santa Cruz Biotechnology) were performed. Histological images of the stained tissues were acquired using a microscope (Leica, Buffalo Grove, IL, USA) equipped with a CCD camera.

### Multiphoton second harmonic generation microscopy and image analysis

Collagen second harmonic generation (SHG) images were collected using a two-photon Zeiss LSM 7 MP microscope (Carl Zeiss, Maple Grove, MN, USA) with a 20 × lens to assess the degree of collagen matrix remodeling. A Ti:sapphire laser (Chameleon, Coherent) was used. The excitation wavelength was 930 nm. The collagen SHG signal from the H&E-stained slides was collected using a 420-480 nm narrow bandpass emission filter. The same laser power and detector gain settings were used when acquiring all images. Three independent experiments were conducted for samples from primary tumors of both the non-silencing shRNA (control) group and the inducible LOXL4 shRNA (shLOXL4) group. Different regions were analyzed for each independent experiment. A MATLAB (MathWorks, Natick, MA, USA) script was written to fit image cross-sections with a spatial correlation function. The Curvelet Transform denoising FIbeR Extraction algorithm (CT-FIRE, LOCI, University of Wisconsin, Madison, WI, USA), an open-source collagen analysis program, was used to automatically analyze individual fiber metrics such as length, width, and straightness, in the images.

### Analysis of the BreastMark dataset

A public online tool, BreastMark, was used to examine the prognostic value of the putative genes in breast cancer patients. This database integrates gene expression and survival data from 26 datasets generated with 12 different microarray platforms and corresponds to approximately 17,000 genes in up to 4,738 samples. Overall survival (OS) was analyzed, and the median was used to dichotomize the data [31]. Median survival was evaluated in high and low LOXL4, collagen I, and collagen IV expression groups; the high expression group included the 25% of samples with the highest expression, and the low expression group included the 25% of samples with the lowest expression. Survival curves were generated based on Kaplan-Meier estimates; the log-rank *p* value with one degree of freedom was used to identify differences in survival, and hazard ratios (HR) with 95% confidence intervals were computed using Cox regression analysis. A hazard ratio of greater than one indicates that the marker was associated with poor prognosis, while a ratio of less than one means that it was associated with good prognosis.

### Statistical analysis

Mean values ± standard deviations for all data were calculated for the results of at least three independent experiments and were statistically evaluated using analysis of variance (ANOVA) and paired *t-*tests. Mann-Whitney U test was used for statistical analysis of the *in vivo* BLI lung images because the shLOXL4 population had larger values than the control population. For all tests, *p*-values of less than 0.05 were considered significant.

## SUPPLEMENTARY MATERIALS FIGURES



## Abbreviations

LOXL4: lysyl oxidase (LOX)-like 4
TNBC: triple-negative breast cancer
ECM: extracellular matrix
SHG: second harmonic generation
PLOD: lysine hydroxylase
P4HA: prolyl 4-hydroxylase subunit alpha
OS: overall survival
