# Supplementary material for: LOXL4 knockdown enhances tumor growth and lung metastasis through collagen-dependent extracellular matrix changes in triple-negative breast cancer
Source: Oncotarget. 2017 Jan 2;8(7):11977–89. doi: 10.18632/oncotarget.14450 (PMC5355319; doi:10.18632/oncotarget.14450)
Supplement: Supplementary file 1 [file oncotarget-08-11977-s001.pdf]

LOXL4 knockdown enhances tumor growth and lung metastasis through collagen-dependent extracellular matrix changes in triple-negative breast cancer

SUPPLEMENTARY FIGURES

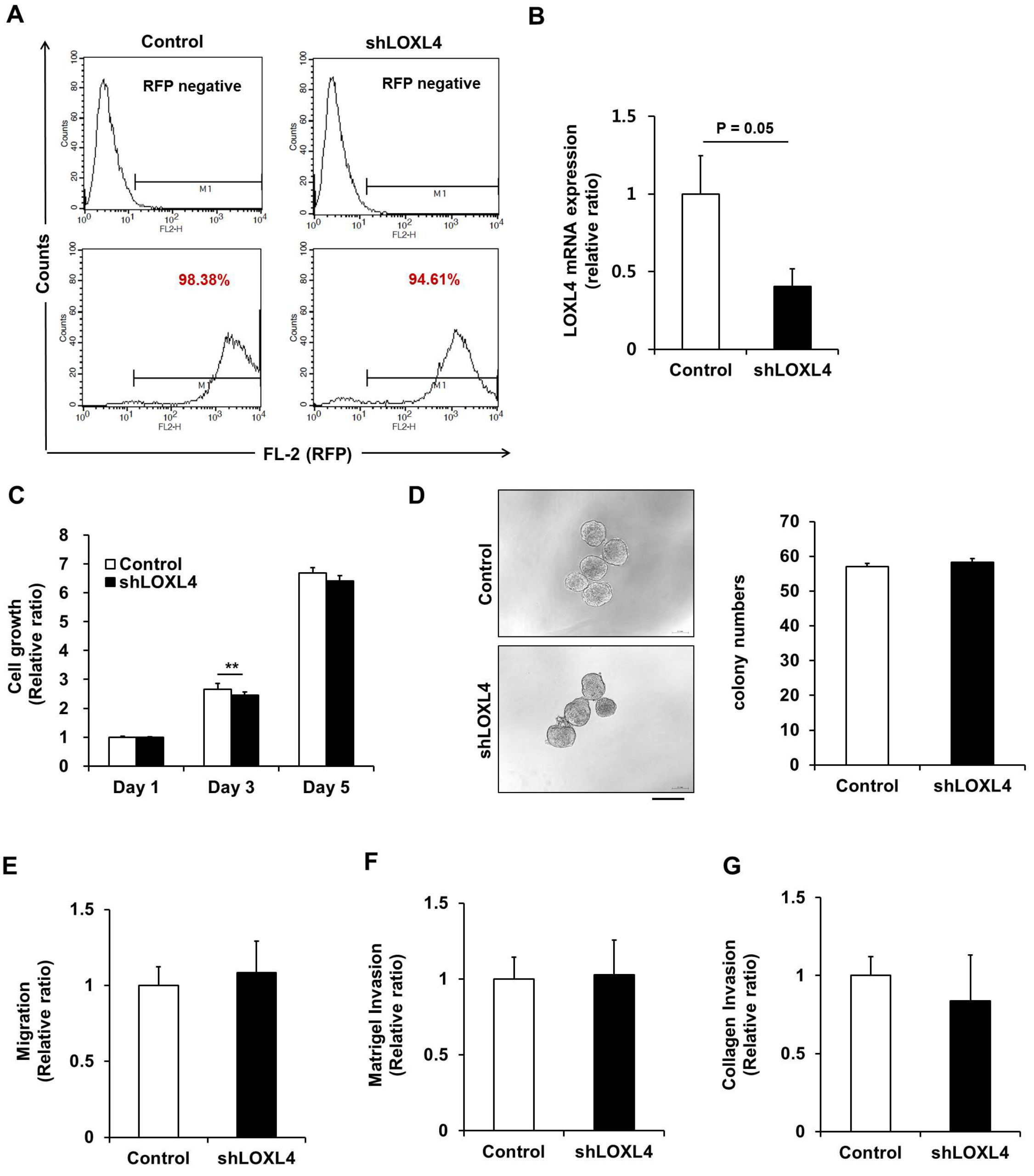

Supplementary Figure 1: Establishment and characterization of LOXL4 knockdown MCF-7 cells. A. Flow cytometric analysis of the percentage of RFP-positive control and LOXL4-knockdown MCF-7 cells. **B.** Quantitative real-time RT-PCR of LOXL4. **C.** MTT assay for analysis of cell proliferation. **D.** Single cell colony formation assay. **E.** Trans-well migration assay for the analysis of cell migratory capacity. **F** and **G.** Trans-well invasion assays for the analysis of cell invasion capacity. All experiments were performed at least in triplicate; means ± standard deviation of all experiments are shown. *$P < 0.05$. **$P < 0.001$. Scale bar: 200 µm.

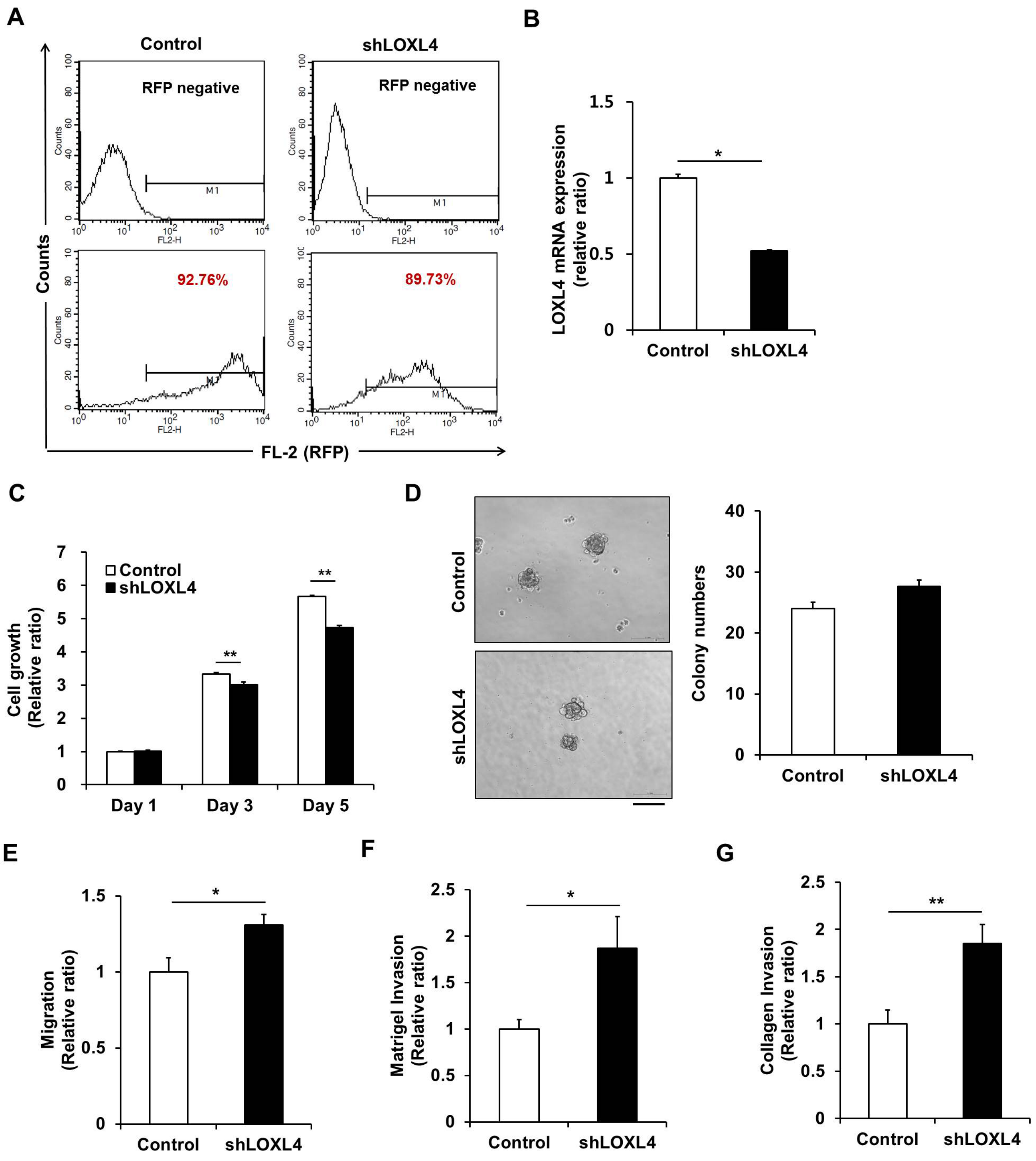

Supplementary Figure 2: Establishment and characterization of LOXL4 knockdown BT-549 cells. A. Flow cytometric analysis of the percentage of RFP-positive control and LOXL4-knockdown BT-549 cells. **B.** Quantitative real-time RT-PCR of LOXL4. **C.** MTT assay for analysis of cell proliferation. **D.** Single cell colony formation assay. **E.** Trans-well migration assay for the analysis of cell migratory capacity. **F** and **G.** Trans-well invasion assays for the analysis of cell invasion capacity. All the experiments were performed at least in triplicate; means ± standard deviation of all experiments are shown. **P* < 0.05. ***P* < 0.001. Scale bar: 100 μm.

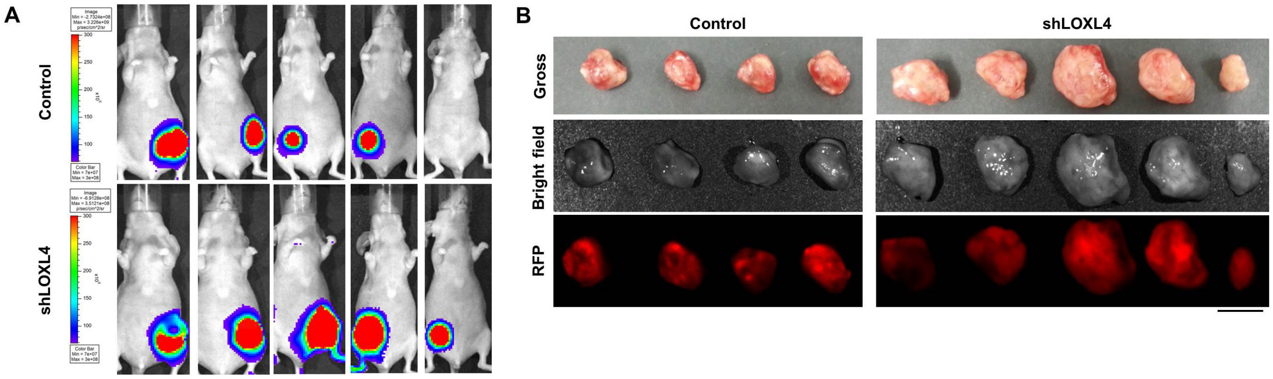

Supplementary Figure 3: LOXL4 knockdown increased primary MDA-MB-231 breast cancer cell tumor growth. **A.** Bioluminescence images of tumors obtained with the IVIS system on day 41 after cancer cell injection. **B.** Images of final lung gross appearance and doxycycline-induced RFP in primary tumors. Scale bar: 1 cm. Control and shLOXL4, n = 4 and 5, respectively.

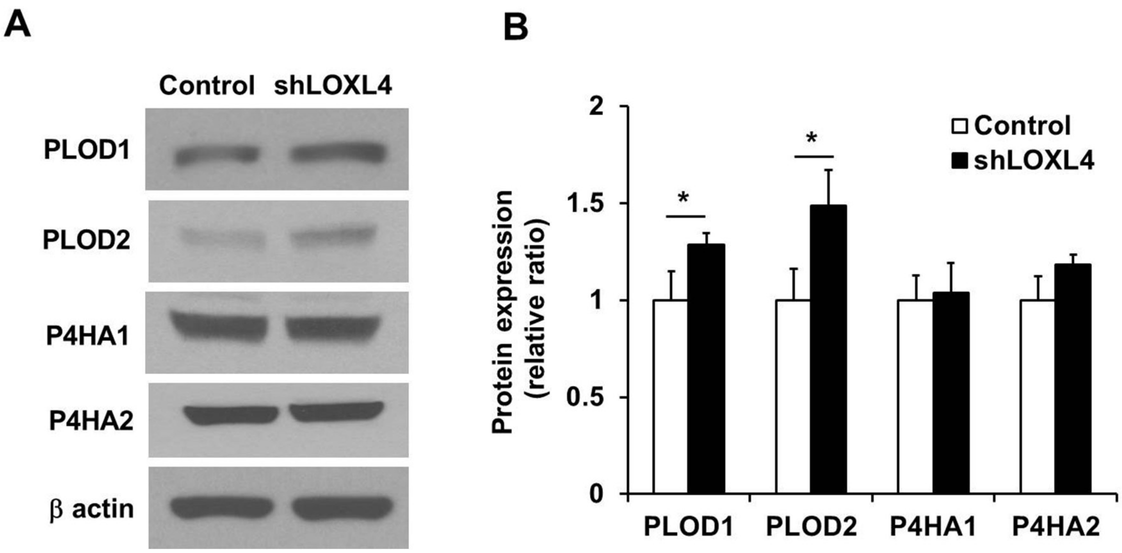

Supplementary Figure 4: PLOD1, PLOD2, P4HA1, and P4HA2 expression in LOXL4 knockdown MDA-MB-231 cells. **A.** Western blotting analysis of PLOD1-2 and P4HA1-2 protein expression. **B.** Densitometric quantification of PLOD1-2 and P4HA1-2 expression.
